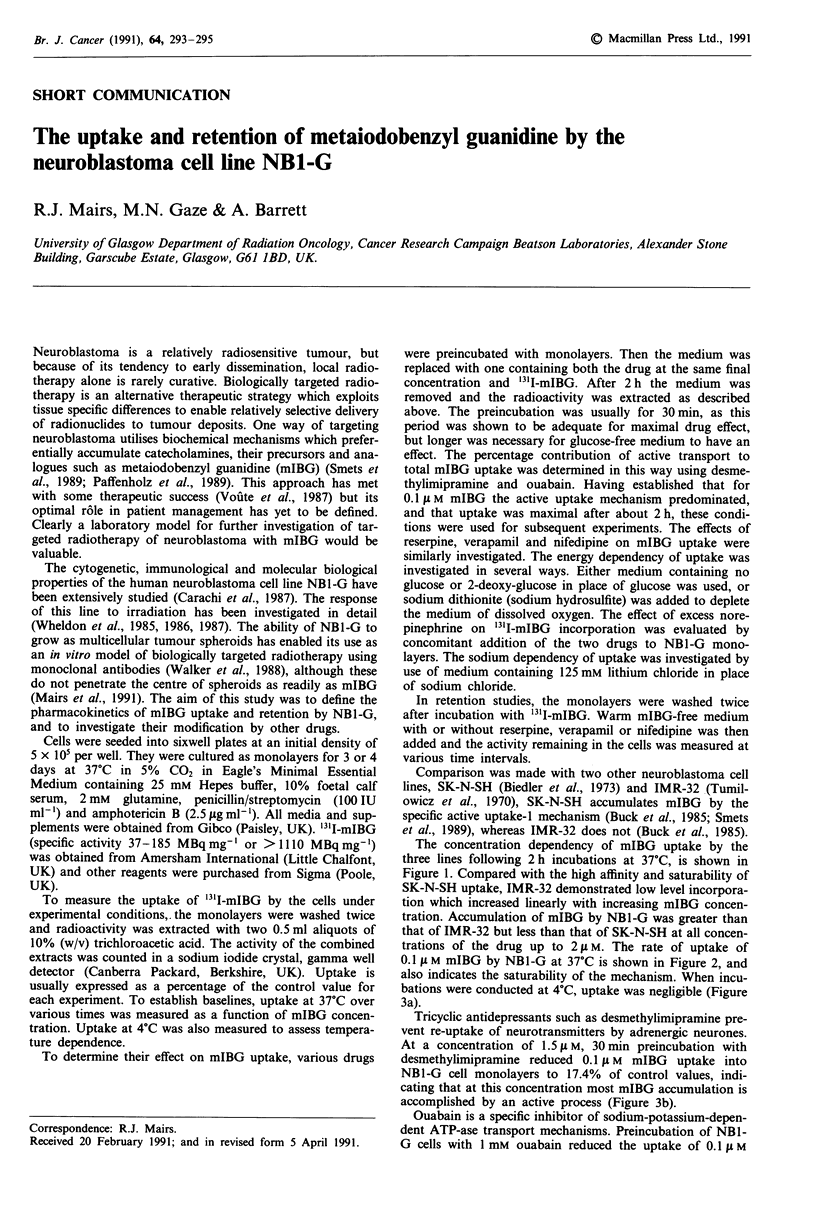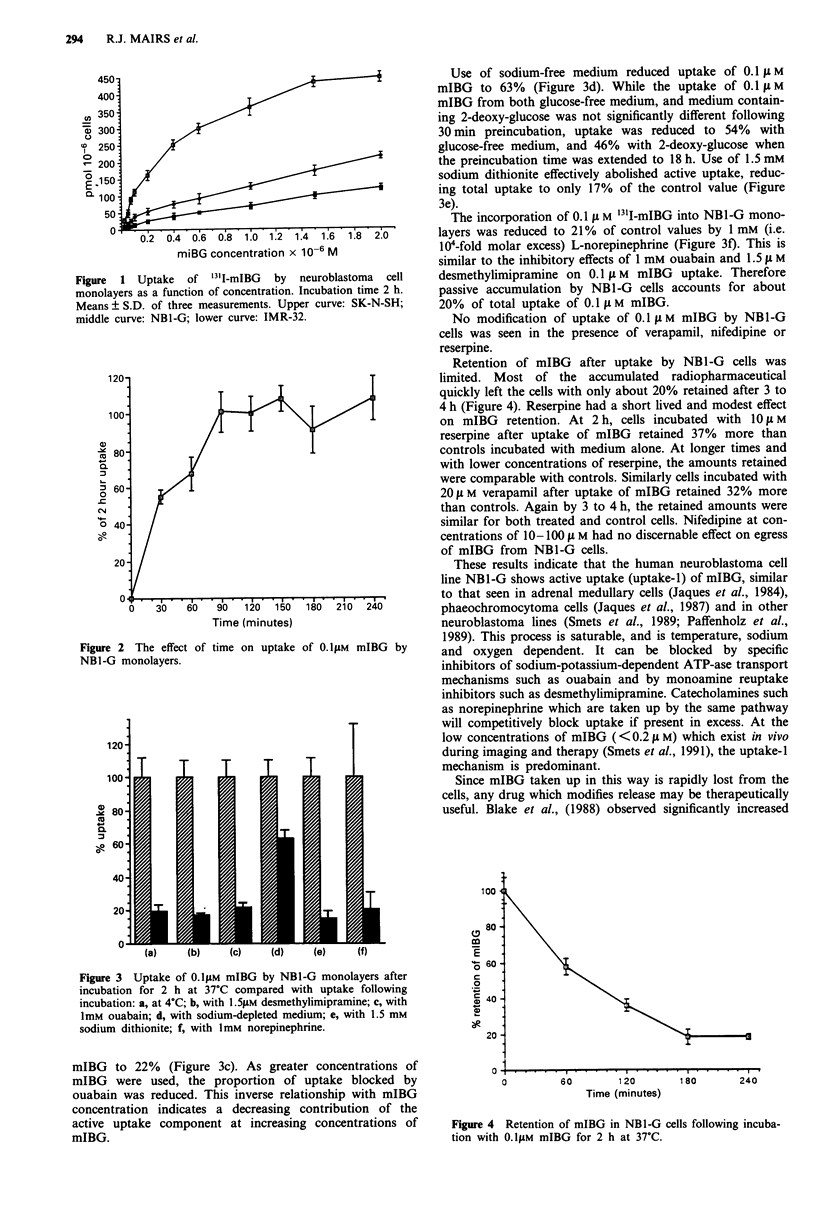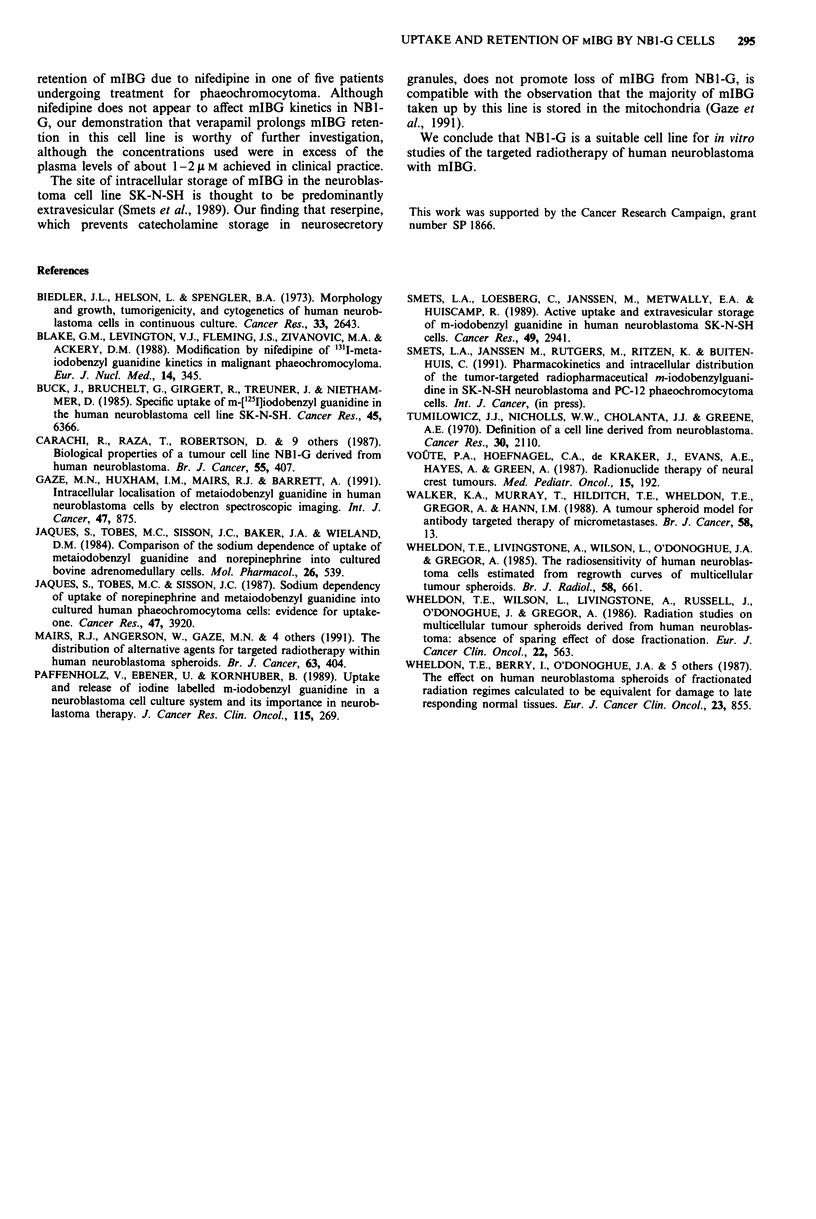# The uptake and retention of metaiodobenzyl guanidine by the neuroblastoma cell line NB1-G.

**DOI:** 10.1038/bjc.1991.294

**Published:** 1991-08

**Authors:** R. J. Mairs, M. N. Gaze, A. Barrett

**Affiliations:** University of Glasgow Department of Radiation Oncology, Cancer Research Campaign Beatson Laboratories, UK.


*Br. J. Cancer* (1991), **64**, 293–295 © Macmillan Press Ltd., 1991

SHORT COMMUNICATION

### The uptake and retention of metaiodobenzyl guanidine by the neuroblastoma cell line NB1-G

R.J. Mairs, M.N. Gaze & A. Barrett

*University of Glasgow Department of Radiation Oncology, Cancer Research Campaign Beatson Laboratories, Alexander Stone Building, Garscube Estate, Glasgow, G61 1BD, UK.*

Neuroblastoma is a relatively radiosensitive tumour, but because of its tendency to early dissemination, local radiotherapy alone is rarely curative. Biologically targeted radiotherapy is an alternative therapeutic strategy which exploits tissue specific differences to enable relatively selective delivery of radionuclides to tumour deposits. One way of targeting neuroblastoma utilises biochemical mechanisms which preferentially accumulate catecholamines, their precursors and analogues such as metaiodobenzyl guanidine (mIBG) (Smets *et al.*, 1989; Paffenholz *et al.*, 1989). This approach has met with some therapeutic success (Voûte *et al.*, 1987) but its optimal rôle in patient management has yet to be defined. Clearly a laboratory model for further investigation of targeted radiotherapy of neuroblastoma with mIBG would be valuable.

The cytogenetic, immunological and molecular biological properties of the human neuroblastoma cell line NB1-G have been extensively studied (Carachi *et al.*, 1987). The response of this line to irradiation has been investigated in detail (Wheldon *et al.*, 1985, 1986, 1987). The ability of NB1-G to grow as multicellular tumour spheroids has enabled its use as an *in vitro* model of biologically targeted radiotherapy using monoclonal antibodies (Walker *et al.*, 1988), although these do not penetrate the centre of spheroids as readily as mIBG (Mairs *et al.*, 1991). The aim of this study was to define the pharmacokinetics of mIBG uptake and retention by NB1-G, and to investigate their modification by other drugs.

Cells were seeded into sixwell plates at an initial density of $5 \times 10^5$ per well. They were cultured as monolayers for 3 or 4 days at 37°C in 5% $CO_2$ in Eagle's Minimal Essential Medium containing 25 mM Hepes buffer, 10% foetal calf serum, 2 mM glutamine, penicillin/streptomycin (100 IU $ml^{-1}$) and amphotericin B (2.5 $\mu g\,ml^{-1}$). All media and supplements were obtained from Gibco (Paisley, UK). $^{131}$I-mIBG (specific activity 37–185 $MBq\,mg^{-1}$ or $>$1110 $MBq\,mg^{-1}$) was obtained from Amersham International (Little Chalfont, UK) and other reagents were purchased from Sigma (Poole, UK).

To measure the uptake of $^{131}$I-mIBG by the cells under experimental conditions,. the monolayers were washed twice and radioactivity was extracted with two 0.5 ml aliquots of 10% (w/v) trichloroacetic acid. The activity of the combined extracts was counted in a sodium iodide crystal, gamma well detector (Canberra Packard, Berkshire, UK). Uptake is usually expressed as a percentage of the control value for each experiment. To establish baselines, uptake at 37°C over various times was measured as a function of mIBG concentration. Uptake at 4°C was also measured to assess temperature dependence.

To determine their effect on mIBG uptake, various drugs were preincubated with monolayers. Then the medium was replaced with one containing both the drug at the same final concentration and $^{131}$I-mIBG. After 2 h the medium was removed and the radioactivity was extracted as described above. The preincubation was usually for 30 min, as this period was shown to be adequate for maximal drug effect, but longer was necessary for glucose-free medium to have an effect. The percentage contribution of active transport to total mIBG uptake was determined in this way using desmethylimipramine and ouabain. Having established that for 0.1 $\mu$M mIBG the active uptake mechanism predominated, and that uptake was maximal after about 2 h, these conditions were used for subsequent experiments. The effects of reserpine, verapamil and nifedipine on mIBG uptake were similarly investigated. The energy dependency of uptake was investigated in several ways. Either medium containing no glucose or 2-deoxy-glucose in place of glucose was used, or sodium dithionite (sodium hydrosulfite) was added to deplete the medium of dissolved oxygen. The effect of excess norepinephrine on $^{131}$I-mIBG incorporation was evaluated by concomitant addition of the two drugs to NB1-G monolayers. The sodium dependency of uptake was investigated by use of medium containing 125 mM lithium chloride in place of sodium chloride.

In retention studies, the monolayers were washed twice after incubation with $^{131}$I-mIBG. Warm mIBG-free medium with or without reserpine, verapamil or nifedipine was then added and the activity remaining in the cells was measured at various time intervals.

Comparison was made with two other neuroblastoma cell lines, SK-N-SH (Biedler *et al.*, 1973) and IMR-32 (Tumilowicz *et al.*, 1970), SK-N-SH accumulates mIBG by the specific active uptake-1 mechanism (Buck *et al.*, 1985; Smets *et al.*, 1989), whereas IMR-32 does not (Buck *et al.*, 1985).

The concentration dependency of mIBG uptake by the three lines following 2 h incubations at 37°C, is shown in Figure 1. Compared with the high affinity and saturability of SK-N-SH uptake, IMR-32 demonstrated low level incorporation which increased linearly with increasing mIBG concentration. Accumulation of mIBG by NB1-G was greater than that of IMR-32 but less than that of SK-N-SH at all concentrations of the drug up to 2 $\mu$M. The rate of uptake of 0.1 $\mu$M mIBG by NB1-G at 37°C is shown in Figure 2, and also indicates the saturability of the mechanism. When incubations were conducted at 4°C, uptake was negligible (Figure 3a).

Tricyclic antidepressants such as desmethylimipramine prevent re-uptake of neurotransmitters by adrenergic neurones. At a concentration of 1.5 $\mu$M, 30 min preincubation with desmethylimipramine reduced 0.1 $\mu$M mIBG uptake into NB1-G cell monolayers to 17.4% of control values, indicating that at this concentration most mIBG accumulation is accomplished by an active process (Figure 3b).

Ouabain is a specific inhibitor of sodium-potassium-dependent ATP-ase transport mechanisms. Preincubation of NB1-G cells with 1 mM ouabain reduced the uptake of 0.1 $\mu$M

Correspondence: R.J. Mairs.
Received 20 February 1991; and in revised form 5 April 1991.

**Figure 1** Uptake of $^{131}$I-mIBG by neuroblastoma cell monolayers as a function of concentration. Incubation time 2 h. Means ± S.D. of three measurements. Upper curve: SK-N-SH; middle curve: NB1-G; lower curve: IMR-32.

**Figure 2** The effect of time on uptake of 0.1μM mIBG by NB1-G monolayers.

**Figure 3** Uptake of 0.1μM mIBG by NB1-G monolayers after incubation for 2 h at 37°C compared with uptake following incubation: **a**, at 4°C; **b**, with 1.5μM desmethylimipramine; **c**, with 1mM ouabain; **d**, with sodium-depleted medium; **e**, with 1.5 mM sodium dithionite; **f**, with 1mM norepinephrine.

mIBG to 22% (Figure 3c). As greater concentrations of mIBG were used, the proportion of uptake blocked by ouabain was reduced. This inverse relationship with mIBG concentration indicates a decreasing contribution of the active uptake component at increasing concentrations of mIBG.

Use of sodium-free medium reduced uptake of 0.1 μM mIBG to 63% (Figure 3d). While the uptake of 0.1 μM mIBG from both glucose-free medium, and medium containing 2-deoxy-glucose was not significantly different following 30 min preincubation, uptake was reduced to 54% with glucose-free medium, and 46% with 2-deoxy-glucose when the preincubation time was extended to 18 h. Use of 1.5 mM sodium dithionite effectively abolished active uptake, reducing total uptake to only 17% of the control value (Figure 3e).

The incorporation of 0.1 μM $^{131}$I-mIBG into NB1-G monolayers was reduced to 21% of control values by 1 mM (i.e. $10^4$-fold molar excess) L-norepinephrine (Figure 3f). This is similar to the inhibitory effects of 1 mM ouabain and 1.5 μM desmethylimipramine on 0.1 μM mIBG uptake. Therefore passive accumulation by NB1-G cells accounts for about 20% of total uptake of 0.1 μM mIBG.

No modification of uptake of 0.1 μM mIBG by NB1-G cells was seen in the presence of verapamil, nifedipine or reserpine.

Retention of mIBG after uptake by NB1-G cells was limited. Most of the accumulated radiopharmaceutical quickly left the cells with only about 20% retained after 3 to 4 h (Figure 4). Reserpine had a short lived and modest effect on mIBG retention. At 2 h, cells incubated with 10 μM reserpine after uptake of mIBG retained 37% more than controls incubated with medium alone. At longer times and with lower concentrations of reserpine, the amounts retained were comparable with controls. Similarly cells incubated with 20 μM verapamil after uptake of mIBG retained 32% more than controls. Again by 3 to 4 h, the retained amounts were similar for both treated and control cells. Nifedipine at concentrations of 10–100 μM had no discernable effect on egress of mIBG from NB1-G cells.

These results indicate that the human neuroblastoma cell line NB1-G shows active uptake (uptake-1) of mIBG, similar to that seen in adrenal medullary cells (Jaques *et al.*, 1984), phaeochromocytoma cells (Jaques *et al.*, 1987) and in other neuroblastoma lines (Smets *et al.*, 1989; Paffenholz *et al.*, 1989). This process is saturable, and is temperature, sodium and oxygen dependent. It can be blocked by specific inhibitors of sodium-potassium-dependent ATP-ase transport mechanisms such as ouabain and by monoamine reuptake inhibitors such as desmethylimipramine. Catecholamines such as norepinephrine which are taken up by the same pathway will competitively block uptake if present in excess. At the low concentrations of mIBG ($<0.2$ μM) which exist *in vivo* during imaging and therapy (Smets *et al.*, 1991), the uptake-1 mechanism is predominant.

Since mIBG taken up in this way is rapidly lost from the cells, any drug which modifies release may be therapeutically useful. Blake *et al.*, (1988) observed significantly increased

**Figure 4** Retention of mIBG in NB1-G cells following incubation with 0.1μM mIBG for 2 h at 37°C.

retention of mIBG due to nifedipine in one of five patients undergoing treatment for phaeochromocytoma. Although nifedipine does not appear to affect mIBG kinetics in NB1-G, our demonstration that verapamil prolongs mIBG retention in this cell line is worthy of further investigation, although the concentrations used were in excess of the plasma levels of about $1-2\,\mu$M achieved in clinical practice.

The site of intracellular storage of mIBG in the neuroblastoma cell line SK-N-SH is thought to be predominantly extravesicular (Smets *et al.*, 1989). Our finding that reserpine, which prevents catecholamine storage in neurosecretory granules, does not promote loss of mIBG from NB1-G, is compatible with the observation that the majority of mIBG taken up by this line is stored in the mitochondria (Gaze *et al.*, 1991).

We conclude that NB1-G is a suitable cell line for *in vitro* studies of the targeted radiotherapy of human neuroblastoma with mIBG.

This work was supported by the Cancer Research Campaign, grant number SP 1866.

### References

BIEDLER, J.L., HELSON, L. & SPENGLER, B.A. (1973). Morphology and growth, tumorigenicity, and cytogenetics of human neuroblastoma cells in continuous culture. *Cancer Res.*, **33**, 2643.

BLAKE, G.M., LEVINGTON, V.J., FLEMING, J.S., ZIVANOVIC, M.A. & ACKERY, D.M. (1988). Modification by nifedipine of $^{131}$I-metaiodobenzyl guanidine kinetics in malignant phaeochromocyloma. *Eur. J. Nucl. Med.*, **14**, 345.

BUCK, J., BRUCHELT, G., GIRGERT, R., TREUNER, J. & NIETHAMMER, D. (1985). Specific uptake of m-[$^{125}$I]iodobenzyl guanidine in the human neuroblastoma cell line SK-N-SH. *Cancer Res.*, **45**, 6366.

CARACHI, R., RAZA, T., ROBERTSON, D. & 9 others (1987). Biological properties of a tumour cell line NB1-G derived from human neuroblastoma. *Br. J. Cancer*, **55**, 407.

GAZE, M.N., HUXHAM, I.M., MAIRS, R.J. & BARRETT, A. (1991). Intracellular localisation of metaiodobenzyl guanidine in human neuroblastoma cells by electron spectroscopic imaging. *Int. J. Cancer*, **47**, 875.

JAQUES, S., TOBES, M.C., SISSON, J.C., BAKER, J.A. & WIELAND, D.M. (1984). Comparison of the sodium dependence of uptake of metaiodobenzyl guanidine and norepinephrine into cultured bovine adrenomedullary cells. *Mol. Pharmacol.*, **26**, 539.

JAQUES, S., TOBES, M.C. & SISSON, J.C. (1987). Sodium dependency of uptake of norepinephrine and metaiodobenzyl guanidine into cultured human phaeochromocytoma cells: evidence for uptake-one. *Cancer Res.*, **47**, 3920.

MAIRS, R.J., ANGERSON, W., GAZE, M.N. & 4 others (1991). The distribution of alternative agents for targeted radiotherapy within human neuroblastoma spheroids. *Br. J. Cancer*, **63**, 404.

PAFFENHOLZ, V., EBENER, U. & KORNHUBER, B. (1989). Uptake and release of iodine labelled m-iodobenzyl guanidine in a neuroblastoma cell culture system and its importance in neuroblastoma therapy. *J. Cancer Res. Clin. Oncol.*, **115**, 269.

SMETS, L.A., LOESBERG, C., JANSSEN, M., METWALLY, E.A. & HUISCAMP, R. (1989). Active uptake and extravesicular storage of m-iodobenzyl guanidine in human neuroblastoma SK-N-SH cells. *Cancer Res.*, **49**, 2941.

SMETS, L.A., JANSSEN M., RUTGERS, M., RITZEN, K. & BUITENHUIS, C. (1991). Pharmacokinetics and intracellular distribution of the tumor-targeted radiopharmaceutical *m*-iodobenzylguanidine in SK-N-SH neuroblastoma and PC-12 phaeochromocytoma cells. *Int. J. Cancer*, (in press).

TUMILOWICZ, J.J., NICHOLLS, W.W., CHOLANTA, J.J. & GREENE, A.E. (1970). Definition of a cell line derived from neuroblastoma. *Cancer Res.*, **30**, 2110.

VOÛTE, P.A., HOEFNAGEL, C.A., de KRAKER, J., EVANS, A.E., HAYES, A. & GREEN, A. (1987). Radionuclide therapy of neural crest tumours. *Med. Pediatr. Oncol.*, **15**, 192.

WALKER, K.A., MURRAY, T., HILDITCH, T.E., WHELDON, T.E., GREGOR, A. & HANN, I.M. (1988). A tumour spheroid model for antibody targeted therapy of micrometastases. *Br. J. Cancer*, **58**, 13.

WHELDON, T.E., LIVINGSTONE, A., WILSON, L., O'DONOGHUE, J.A. & GREGOR, A. (1985). The radiosensitivity of human neuroblastoma cells estimated from regrowth curves of multicellular tumour spheroids. *Br. J. Radiol.*, **58**, 661.

WHELDON, T.E., WILSON, L., LIVINGSTONE, A., RUSSELL, J., O'DONOGHUE, J. & GREGOR, A. (1986). Radiation studies on multicellular tumour spheroids derived from human neuroblastoma: absence of sparing effect of dose fractionation. *Eur. J. Cancer Clin. Oncol.*, **22**, 563.

WHELDON, T.E., BERRY, I., O'DONOGHUE, J.A. & 5 others (1987). The effect on human neuroblastoma spheroids of fractionated radiation regimes calculated to be equivalent for damage to late responding normal tissues. *Eur. J. Cancer Clin. Oncol.*, **23**, 855.